# A new β-octa­molybdate(VI) salt based on 1,4-bis­(2-methyl-1*H*-imidazol-1-yl)butane

**DOI:** 10.1107/S1600536808035022

**Published:** 2008-11-08

**Authors:** Shun-Li Li, Ke Tan

**Affiliations:** a Department of Chemistry, Northeast Normal University, Changchun 130024, People’s Republic of China; b Biological Scientific and Technical College, Changchun University, Changchun 130022, People’s Republic of China

## Abstract

The title compound, bis­[2,2′-dimethyl-3,3′-(butane-1,4-di­yl)diimidazol-1-ium] β-octa­molybdate(VI), (C_12_H_20_N_4_)_2_[Mo_8_O_26_], was produced by hydro­thermal reaction of an acidified aqueous solution of Na_2_MoO_4_ and 1,4-bis­(2-methyl-1*H*-imidazol-1-yl)butane (hereafter *L*). The structure of the title compound consists of the β-octa­molybdate anions having a center of symmetry, and protonated [H_2_ *L*]^2+^ cations, which link the β-octa­molybdate anions, generating a supra­molecular chain *via* hydrogen bonds.

## Related literature

For the applications of polyoxometalates (POMs) chemistry, see: Kozhevnikov (1998 ▶); Rhule *et al.* (1998 ▶); Li *et al.* (2007 ▶). For the coordination ability of polyoxometalates with different transition-metal organic units, see: Hagrman *et al.* (1997 ▶); Li *et al.* (2008 ▶). For the introduction of POMs into coordination polymers for the construction of polymers with desired properties, see: Bu *et al.* (2001 ▶); Wu *et al.* (2002 ▶).
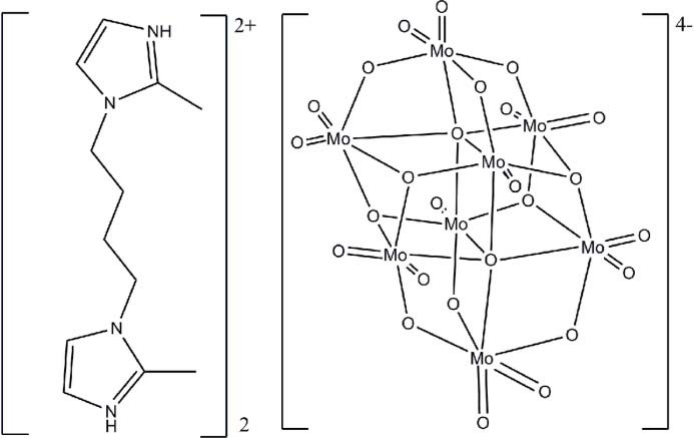


## Experimental

### 

#### Crystal data

(C_12_H_20_N_4_)_2_[Mo_8_O_26_]
*M* *_r_* = 1624.16
Triclinic, 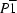

*a* = 10.5680 (3) Å
*b* = 11.4890 (5) Å
*c* = 11.5600 (8) Å
α = 60.7770 (10)°
β = 68.1550 (10)°
γ = 70.4000 (10)°
*V* = 1116.29 (10) Å^3^
*Z* = 1
Mo *K*α radiation
μ = 2.27 mm^−1^
*T* = 293 (2) K
0.27 × 0.26 × 0.20 mm

#### Data collection

Bruker APEX CCD area-detector diffractometer
Absorption correction: multi-scan (*SADABS*; Sheldrick, 1996 ▶) *T*_min_ = 0.49, *T*_max_ = 0.63
6962 measured reflections
5123 independent reflections
3776 reflections with *I* > 2σ(*I*)
*R*_int_ = 0.016

#### Refinement

*R*[*F* ^2^ > 2σ(*F* ^2^)] = 0.032
*wR*(*F* ^2^) = 0.079
*S* = 1.04
5123 reflections
306 parameters
2 restraints
H atoms treated by a mixture of independent and constrained refinement
Δρ_max_ = 0.53 e Å^−3^
Δρ_min_ = −0.71 e Å^−3^

### 

Data collection: *SMART* (Bruker, 1999 ▶); cell refinement: *SAINT* (Bruker, 1999 ▶); data reduction: *SAINT*; program(s) used to solve structure: *SHELXS97* (Sheldrick, 2008 ▶); program(s) used to refine structure: *SHELXL97* (Sheldrick, 2008 ▶); molecular graphics: *SHELXTL-Plus* (Sheldrick, 2008 ▶); software used to prepare material for publication: *SHELXL97*.

## Supplementary Material

Crystal structure: contains datablocks global, I. DOI: 10.1107/S1600536808035022/bg2208sup1.cif

Structure factors: contains datablocks I. DOI: 10.1107/S1600536808035022/bg2208Isup2.hkl

Additional supplementary materials:  crystallographic information; 3D view; checkCIF report

## Figures and Tables

**Table 1:** Hydrogen-bond geometry (Å, °)

*D*—H⋯*A*	*D*—H	H⋯*A*	*D*⋯*A*	*D*—H⋯*A*
N2—H2*N*⋯O9^i^	0.87 (3)	2.37 (4)	3.031 (5)	134 (4)
N2—H2*N*⋯O10^i^	0.87 (3)	2.20 (2)	3.001 (5)	153 (5)
N4—H4*N*⋯O12^ii^	0.86 (3)	2.39 (4)	3.052 (5)	134 (4)
N4—H4*N*⋯O4^iii^	0.86 (3)	2.12 (3)	2.873 (4)	146 (5)

Symmetry codes: (i) 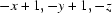; (ii) 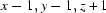; (iii) 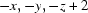.

**Fig. 1.**
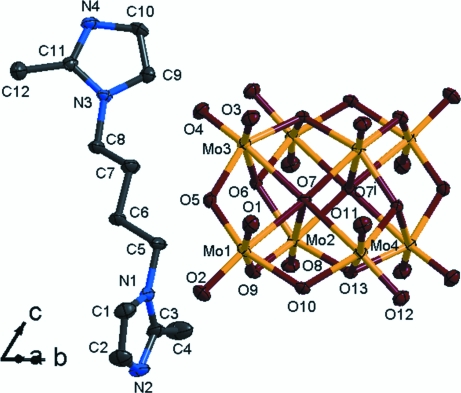
A view of the molecule of (I). Displacement ellipsoids are drawn at the 30% probability level. All crystallographic related oxygen atoms are unlabled for clarity. Symmetry code: (i) 1 - x, 1 - y, -z.

**Fig. 2.**
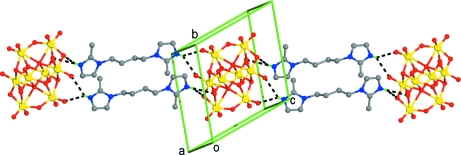
Ball-stick representation of the one-dimensional supramolecular structure of (I).

## supplementary crystallographic information

### Comment

Polyoxometalates (POMs), a unique class of metal-oxide clusters, have many properties that make them attractive for applications in catalysis, biology, magnetism, optics, medicine, *etc* (Kozhevnikov, 1998; Rhule *et al.*, 1998; Li *et al.*, 2007). In recent times a remarkable approach to the construction of multifunctional materials is being realized exploting the ability of polyoxometalates to coordinate to different transition-metal organic units (Hagrman *et al.*, 1997; Li *et al.*, 2008). The POMs, acting as unusual inorganic ligands are introduced into a variety of POM-based coordination polymers with desired properties (Bu *et al.*, 2001; Wu *et al.*, 2002). During our ongoing studies of related materials, we obtained the title compound, (I), and present its crystal structure here.

The asymmetric unit of compound (I) contains a complete (C_12_H_20_N_4_^2+^) cation (hereafter [H_2_L]^2+^) and half a [Mo_8_O_26_]^4-^ anion. The complete [Mo_8_O_26_]^4-^ moiety is generated from the asymmetric unit atoms by a crystallographic inversion center (Fig. 1). It consists of eight edge-sharing MoO_6_ octahedra and displays the characteristic β-octamolybdate arrangement. Each protonated [H_2_L]^2+^ cation donates two N—H···O hydrogen bonds to two terminal oxygen atoms from one [Mo_8_O_26_]^4-^ anion and two ones to two bridging oxygen atoms from the other [Mo_8_O_26_]^4-^ anion. So each [Mo_8_O_26_]^4-^ anion joins four protonated [H_2_L]^2+^ cations (see the hydrogen bonding table for numerical values) to generate a one-dimensional supramolecular double-chain structure (Fig. 2).

### Experimental

A mixture of Na_2_MoO_4_.2H_2_O (0.242 g, 1.0 mmol) and *L* (0.218 g, 1.0 mmol) in water (10 ml) was adjusted with HCl (2*M*) to pH = 3. Then the mixture was placed in a 23 ml Teflon-lined autoclave and kept under autogenous pressure at 150 °C for 2 days. After the mixture was cooled to room temperature at 10°C.h^-1^, colorless crystals of the title compound were obtained.

### Refinement

All H atoms on C atoms were positioned geometrically and refined as riding atoms, with C—H = 0.93 - 0.97 Å, and *U*_iso_=1.2*U*_eq_ (C). The H atoms of N2 and N4 were located in a difference Fourier map and then refined isotropically, with restrained N-H (0.87 (3)Å) and *U*_iso_=1.5*U*_eq_ (N).

### Crystal data

**Table:**

(C_12_H_20_N_4_)_2_[Mo_8_O_26_]	*Z* = 1
*M**_r_* = 1624.16	*F*_000_ = 784
Triclinic, *P*1	*D*_x_ = 2.416 Mg m^−^^3^
Hall symbol: -p1	Mo *K*α radiation λ = 0.71069 Å
*a* = 10.5680 (3) Å	Cell parameters from 879 reflections
*b* = 11.4890 (5) Å	θ = 2.1–28.3º
*c* = 11.5600 (8) Å	µ = 2.27 mm^−^^1^
α = 60.7770 (10)º	*T* = 293 (2) K
β = 68.1550 (10)º	Block, colorless
γ = 70.4000 (10)º	0.27 × 0.26 × 0.20 mm
*V* = 1116.29 (10) Å^3^	

### Data collection

**Table:**

Bruker APEX CCD area-detector diffractometer	5123 independent reflections
Radiation source: fine-focus sealed tube	3776 reflections with *I* > 2σ(*I*)
Monochromator: graphite	*R*_int_ = 0.016
*T* = 293(2) K	θ_max_ = 28.3º
ω scans	θ_min_ = 2.1º
Absorption correction: multi-scan(SADABS; Sheldrick, 1996)	*h* = −13→13
*T*_min_ = 0.49, *T*_max_ = 0.63	*k* = −8→15
6962 measured reflections	*l* = −12→15

### Refinement

**Table:**

Refinement on *F*^2^	Secondary atom site location: difference Fourier map
Least-squares matrix: full	Hydrogen site location: inferred from neighbouring sites
*R*[*F*^2^ > 2σ(*F*^2^)] = 0.032	H atoms treated by a mixture of independent and constrained refinement
*wR*(*F*^2^) = 0.079	*w* = 1/[σ^2^(*F*_o_^2^) + (0.0329*P*)^2^] where *P* = (*F*_o_^2^ + 2*F*_c_^2^)/3
*S* = 1.04	(Δ/σ)_max_ = 0.001
5123 reflections	Δρ_max_ = 0.53 e Å^−^^3^
306 parameters	Δρ_min_ = −0.71 e Å^−^^3^
2 restraints	Extinction correction: none
Primary atom site location: structure-invariant direct methods	

### Special details

**Table:**

Geometry. All e.s.d.'s (except the e.s.d. in the dihedral angle between two l.s. planes) are estimated using the full covariance matrix. The cell e.s.d.'s are taken into account individually in the estimation of e.s.d.'s in distances, angles and torsion angles; correlations between e.s.d.'s in cell parameters are only used when they are defined by crystal symmetry. An approximate (isotropic) treatment of cell e.s.d.'s is used for estimating e.s.d.'s involving l.s. planes.
Refinement. Refinement of *F*^2^ against ALL reflections. The weighted *R*-factor *wR* and goodness of fit *S* are based on *F*^2^, conventional *R*-factors *R* are based on *F*, with *F* set to zero for negative *F*^2^. The threshold expression of *F*^2^ > σ(*F*^2^) is used only for calculating *R*-factors(gt) *etc*. and is not relevant to the choice of reflections for refinement. *R*-factors based on *F*^2^ are statistically about twice as large as those based on *F*, and *R*- factors based on ALL data will be even larger.

### Fractional atomic coordinates and isotropic or equivalent isotropic displacement parameters (Å^2^)

**Table:**

	*x*	*y*	*z*	*U*_iso_*/*U*_eq_	
C1	0.4508 (5)	0.2008 (5)	0.1689 (5)	0.0480 (13)	
H1	0.5040	0.1222	0.2199	0.058*	
C2	0.4674 (5)	0.2537 (5)	0.0338 (5)	0.0516 (14)	
H2	0.5345	0.2198	−0.0273	0.062*	
C3	0.2898 (5)	0.3852 (5)	0.1169 (5)	0.0398 (12)	
C4	0.1745 (7)	0.4991 (6)	0.1210 (6)	0.078 (2)	
H4A	0.0929	0.4647	0.1863	0.117*	
H4B	0.1566	0.5524	0.0320	0.117*	
H4C	0.1983	0.5547	0.1475	0.117*	
C5	0.2836 (5)	0.2607 (5)	0.3649 (4)	0.0455 (13)	
H5A	0.2434	0.3477	0.3706	0.055*	
H5B	0.3589	0.2175	0.4099	0.055*	
C6	0.1756 (5)	0.1742 (5)	0.4387 (4)	0.0357 (11)	
H6A	0.2156	0.0864	0.4349	0.043*	
H6B	0.1002	0.2167	0.3940	0.043*	
C7	0.1188 (5)	0.1543 (5)	0.5889 (5)	0.0454 (13)	
H7A	0.1859	0.0893	0.6405	0.055*	
H7B	0.1035	0.2398	0.5943	0.055*	
C8	−0.0135 (5)	0.1044 (5)	0.6487 (4)	0.0380 (11)	
H8A	−0.0808	0.1724	0.5989	0.046*	
H8B	0.0018	0.0228	0.6362	0.046*	
C9	−0.0909 (5)	0.1582 (4)	0.8570 (5)	0.0333 (10)	
H9	−0.0603	0.2401	0.8147	0.040*	
C10	−0.1608 (5)	0.1011 (5)	0.9878 (5)	0.0375 (11)	
H10	−0.1887	0.1351	1.0536	0.045*	
C11	−0.1292 (4)	−0.0326 (4)	0.8885 (4)	0.0305 (10)	
C12	−0.1334 (6)	−0.1486 (5)	0.8684 (5)	0.0504 (14)	
H12A	−0.2059	−0.1234	0.8248	0.076*	
H12B	−0.1511	−0.2244	0.9557	0.076*	
H12C	−0.0458	−0.1735	0.8117	0.076*	
O1	0.8514 (3)	0.1866 (3)	0.4043 (3)	0.0368 (8)	
O2	0.7405 (3)	0.2236 (3)	0.2112 (3)	0.0440 (8)	
O3	0.6495 (3)	0.1219 (3)	0.7129 (3)	0.0382 (8)	
O4	0.3883 (3)	0.1137 (3)	0.7404 (3)	0.0358 (7)	
O5	0.5723 (3)	0.1829 (3)	0.4809 (3)	0.0296 (7)	
O6	0.3415 (3)	0.3836 (3)	0.5375 (3)	0.0242 (6)	
O7	0.6125 (3)	0.3992 (3)	0.4802 (3)	0.0224 (6)	
O8	0.2891 (3)	0.6211 (3)	0.3047 (3)	0.0350 (7)	
O9	0.5161 (3)	0.4268 (3)	0.2790 (3)	0.0280 (6)	
O10	0.7738 (3)	0.4496 (3)	0.2258 (3)	0.0289 (7)	
O11	0.9029 (3)	0.4409 (3)	0.4018 (3)	0.0368 (8)	
O12	0.8404 (3)	0.6795 (3)	0.1956 (3)	0.0368 (7)	
O13	0.5532 (3)	0.6534 (3)	0.2769 (3)	0.0228 (6)	
Mo1	0.71426 (4)	0.27605 (4)	0.33399 (4)	0.02625 (10)	
Mo2	0.42574 (3)	0.52592 (3)	0.37192 (3)	0.02135 (9)	
Mo3	0.51296 (4)	0.21069 (3)	0.64292 (4)	0.02435 (10)	
Mo4	0.77331 (3)	0.54199 (3)	0.32507 (3)	0.02360 (10)	
N1	0.3404 (4)	0.2840 (4)	0.2189 (4)	0.0347 (9)	
N2	0.3664 (4)	0.3676 (4)	0.0030 (4)	0.0434 (10)	
H2N	0.355 (5)	0.420 (4)	−0.079 (2)	0.064*	
N3	−0.0723 (3)	0.0740 (3)	0.7959 (3)	0.0266 (8)	
N4	−0.1830 (4)	−0.0186 (4)	1.0052 (4)	0.0362 (9)	
H4N	−0.220 (5)	−0.079 (4)	1.082 (3)	0.054*	

### Atomic displacement parameters (Å^2^)

**Table:**

	*U*^11^	*U*^22^	*U*^33^	*U*^12^	*U*^13^	*U*^23^
C1	0.042 (3)	0.046 (3)	0.040 (3)	−0.004 (2)	−0.010 (2)	−0.009 (2)
C2	0.042 (3)	0.059 (4)	0.039 (3)	−0.003 (3)	0.007 (2)	−0.025 (3)
C3	0.044 (3)	0.037 (3)	0.028 (2)	−0.013 (2)	0.003 (2)	−0.012 (2)
C4	0.091 (5)	0.053 (4)	0.048 (4)	0.012 (3)	−0.003 (3)	−0.018 (3)
C5	0.056 (3)	0.065 (4)	0.022 (2)	−0.035 (3)	−0.003 (2)	−0.013 (2)
C6	0.039 (3)	0.045 (3)	0.024 (2)	−0.016 (2)	0.002 (2)	−0.016 (2)
C7	0.047 (3)	0.063 (4)	0.028 (3)	−0.028 (3)	0.004 (2)	−0.018 (2)
C8	0.043 (3)	0.050 (3)	0.023 (2)	−0.020 (2)	0.000 (2)	−0.017 (2)
C9	0.036 (3)	0.028 (2)	0.034 (3)	−0.0015 (19)	−0.010 (2)	−0.013 (2)
C10	0.038 (3)	0.048 (3)	0.030 (3)	−0.001 (2)	−0.008 (2)	−0.024 (2)
C11	0.029 (2)	0.035 (3)	0.027 (2)	−0.0091 (19)	−0.0080 (19)	−0.011 (2)
C12	0.064 (4)	0.051 (3)	0.043 (3)	−0.025 (3)	−0.012 (3)	−0.017 (3)
O1	0.0320 (17)	0.0321 (18)	0.0421 (19)	0.0006 (14)	−0.0092 (15)	−0.0167 (15)
O2	0.054 (2)	0.050 (2)	0.0372 (19)	−0.0100 (17)	−0.0053 (16)	−0.0287 (17)
O3	0.0367 (18)	0.0301 (17)	0.0383 (18)	0.0010 (14)	−0.0130 (15)	−0.0093 (14)
O4	0.0330 (17)	0.0286 (17)	0.0356 (18)	−0.0082 (13)	−0.0006 (14)	−0.0100 (14)
O5	0.0331 (16)	0.0244 (15)	0.0319 (16)	−0.0076 (13)	−0.0042 (13)	−0.0139 (13)
O6	0.0260 (15)	0.0217 (14)	0.0251 (15)	−0.0066 (12)	−0.0046 (12)	−0.0100 (12)
O7	0.0222 (14)	0.0224 (14)	0.0195 (14)	−0.0035 (11)	−0.0025 (11)	−0.0087 (12)
O8	0.0335 (17)	0.0356 (18)	0.0318 (17)	−0.0043 (14)	−0.0144 (14)	−0.0081 (14)
O9	0.0282 (16)	0.0340 (17)	0.0226 (15)	−0.0049 (13)	−0.0037 (12)	−0.0149 (13)
O10	0.0288 (16)	0.0342 (17)	0.0223 (15)	−0.0055 (13)	0.0011 (12)	−0.0164 (13)
O11	0.0273 (16)	0.0384 (19)	0.0373 (18)	−0.0035 (14)	−0.0056 (14)	−0.0139 (15)
O12	0.0338 (17)	0.0374 (18)	0.0343 (18)	−0.0156 (14)	−0.0015 (14)	−0.0109 (14)
O13	0.0231 (14)	0.0254 (15)	0.0178 (14)	−0.0056 (12)	−0.0043 (11)	−0.0074 (12)
Mo1	0.0270 (2)	0.0275 (2)	0.02393 (19)	−0.00334 (15)	−0.00188 (15)	−0.01489 (16)
Mo2	0.02117 (18)	0.02459 (19)	0.01723 (18)	−0.00511 (14)	−0.00434 (13)	−0.00779 (14)
Mo3	0.02464 (19)	0.02039 (19)	0.02227 (19)	−0.00412 (14)	−0.00317 (15)	−0.00671 (15)
Mo4	0.01997 (18)	0.0257 (2)	0.02189 (19)	−0.00583 (14)	−0.00081 (14)	−0.00971 (15)
N1	0.041 (2)	0.040 (2)	0.025 (2)	−0.0187 (18)	−0.0054 (17)	−0.0099 (17)
N2	0.047 (2)	0.041 (3)	0.022 (2)	−0.008 (2)	0.0007 (19)	−0.0061 (18)
N3	0.0265 (18)	0.033 (2)	0.0194 (17)	−0.0074 (15)	−0.0018 (14)	−0.0118 (15)
N4	0.035 (2)	0.046 (3)	0.0212 (19)	−0.0169 (19)	0.0011 (17)	−0.0095 (18)

### Geometric parameters (Å, °)

**Table:**

C1—C2	1.334 (7)	C11—C12	1.477 (6)
C1—N1	1.378 (6)	C12—H12A	0.9600
C1—H1	0.9300	C12—H12B	0.9600
C2—N2	1.367 (6)	C12—H12C	0.9600
C2—H2	0.9300	O1—Mo1	1.697 (3)
C3—N1	1.319 (6)	O2—Mo1	1.699 (3)
C3—N2	1.337 (6)	O3—Mo3	1.688 (3)
C3—C4	1.465 (7)	O4—Mo3	1.703 (3)
C4—H4A	0.9600	O5—Mo3	1.892 (3)
C4—H4B	0.9600	O5—Mo1	1.920 (3)
C4—H4C	0.9600	O6—Mo2	1.945 (3)
C5—N1	1.481 (5)	O6—Mo3	2.362 (3)
C5—C6	1.492 (6)	O7—Mo3	2.327 (2)
C5—H5A	0.9700	O7—Mo4	2.350 (3)
C5—H5B	0.9700	O7—Mo2	2.390 (3)
C6—C7	1.535 (6)	O7—Mo1	2.444 (3)
C6—H6A	0.9700	O8—Mo2	1.682 (3)
C6—H6B	0.9700	O9—Mo2	1.750 (3)
C7—C8	1.480 (6)	O9—Mo1	2.296 (3)
C7—H7A	0.9700	O10—Mo4	1.904 (3)
C7—H7B	0.9700	O10—Mo1	1.938 (3)
C8—N3	1.478 (5)	O11—Mo4	1.686 (3)
C8—H8A	0.9700	O12—Mo4	1.700 (3)
C8—H8B	0.9700	O13—Mo2	1.954 (3)
C9—C10	1.337 (6)	O13—Mo4	2.373 (3)
C9—N3	1.382 (5)	Mo2—Mo4^i^	3.2019 (5)
C9—H9	0.9300	Mo2—Mo3^i^	3.2144 (5)
C10—N4	1.377 (6)	Mo4—O6^i^	1.981 (3)
C10—H10	0.9300	N2—H2N	0.87 (3)
C11—N3	1.320 (5)	N4—H4N	0.86 (3)
C11—N4	1.322 (5)		
			
C2—C1—N1	107.4 (4)	O5—Mo1—O7	73.80 (10)
C2—C1—H1	126.3	O10—Mo1—O7	74.23 (10)
N1—C1—H1	126.3	O9—Mo1—O7	69.81 (9)
C1—C2—N2	106.5 (4)	O8—Mo2—O9	104.33 (14)
C1—C2—H2	126.8	O8—Mo2—O6	101.53 (13)
N2—C2—H2	126.8	O9—Mo2—O6	96.72 (12)
N1—C3—N2	106.9 (4)	O8—Mo2—O13	101.33 (13)
N1—C3—C4	128.6 (5)	O9—Mo2—O13	95.61 (12)
N2—C3—C4	124.6 (5)	O6—Mo2—O13	150.39 (11)
C3—C4—H4A	109.5	O8—Mo2—O7^i^	99.39 (13)
C3—C4—H4B	109.5	O9—Mo2—O7^i^	156.26 (12)
H4A—C4—H4B	109.5	O6—Mo2—O7^i^	79.18 (10)
C3—C4—H4C	109.5	O13—Mo2—O7^i^	78.73 (10)
H4A—C4—H4C	109.5	O8—Mo2—O7	175.33 (12)
H4B—C4—H4C	109.5	O9—Mo2—O7	80.34 (11)
N1—C5—C6	112.8 (4)	O6—Mo2—O7	77.54 (10)
N1—C5—H5A	109.0	O13—Mo2—O7	78.15 (10)
C6—C5—H5A	109.0	O7^i^—Mo2—O7	75.94 (10)
N1—C5—H5B	109.0	O8—Mo2—Mo4^i^	90.41 (10)
C6—C5—H5B	109.0	O9—Mo2—Mo4^i^	132.43 (9)
H5A—C5—H5B	107.8	O6—Mo2—Mo4^i^	35.72 (8)
C5—C6—C7	110.9 (4)	O13—Mo2—Mo4^i^	125.92 (8)
C5—C6—H6A	109.5	O7^i^—Mo2—Mo4^i^	47.21 (7)
C7—C6—H6A	109.5	O7—Mo2—Mo4^i^	86.22 (6)
C5—C6—H6B	109.5	O8—Mo2—Mo3^i^	90.58 (10)
C7—C6—H6B	109.5	O9—Mo2—Mo3^i^	131.56 (9)
H6A—C6—H6B	108.1	O6—Mo2—Mo3^i^	125.49 (8)
C8—C7—C6	109.9 (4)	O13—Mo2—Mo3^i^	35.96 (8)
C8—C7—H7A	109.7	O7^i^—Mo2—Mo3^i^	46.33 (7)
C6—C7—H7A	109.7	O7—Mo2—Mo3^i^	86.33 (6)
C8—C7—H7B	109.7	Mo4^i^—Mo2—Mo3^i^	92.286 (13)
C6—C7—H7B	109.7	O3—Mo3—O4	104.79 (15)
H7A—C7—H7B	108.2	O3—Mo3—O5	101.97 (14)
N3—C8—C7	114.0 (4)	O4—Mo3—O5	101.64 (14)
N3—C8—H8A	108.8	O3—Mo3—O13^i^	97.07 (13)
C7—C8—H8A	108.8	O4—Mo3—O13^i^	100.09 (13)
N3—C8—H8B	108.8	O5—Mo3—O13^i^	146.22 (11)
C7—C8—H8B	108.8	O3—Mo3—O7	96.22 (12)
H8A—C8—H8B	107.6	O4—Mo3—O7	158.65 (12)
C10—C9—N3	107.8 (4)	O5—Mo3—O7	77.19 (10)
C10—C9—H9	126.1	O13^i^—Mo3—O7	73.19 (10)
N3—C9—H9	126.1	O3—Mo3—O6	165.01 (12)
C9—C10—N4	106.0 (4)	O4—Mo3—O6	87.24 (12)
C9—C10—H10	127.0	O5—Mo3—O6	83.90 (11)
N4—C10—H10	127.0	O13^i^—Mo3—O6	71.63 (10)
N3—C11—N4	108.0 (4)	O7—Mo3—O6	71.41 (9)
N3—C11—C12	127.0 (4)	O3—Mo3—Mo2^i^	86.46 (11)
N4—C11—C12	125.0 (4)	O4—Mo3—Mo2^i^	135.19 (10)
C11—C12—H12A	109.5	O5—Mo3—Mo2^i^	118.52 (8)
C11—C12—H12B	109.5	O13^i^—Mo3—Mo2^i^	35.10 (7)
H12A—C12—H12B	109.5	O7—Mo3—Mo2^i^	41.34 (6)
C11—C12—H12C	109.5	O6—Mo3—Mo2^i^	78.65 (6)
H12A—C12—H12C	109.5	O11—Mo4—O12	104.90 (15)
H12B—C12—H12C	109.5	O11—Mo4—O10	101.89 (14)
Mo3—O5—Mo1	117.70 (14)	O12—Mo4—O10	101.07 (14)
Mo2—O6—Mo4^i^	109.31 (12)	O11—Mo4—O6^i^	98.62 (13)
Mo2—O6—Mo3	110.72 (12)	O12—Mo4—O6^i^	100.79 (13)
Mo4^i^—O6—Mo3	104.16 (11)	O10—Mo4—O6^i^	144.88 (11)
Mo2^i^—O7—Mo3	92.33 (9)	O11—Mo4—O7	94.31 (12)
Mo2^i^—O7—Mo4	91.22 (10)	O12—Mo4—O7	160.60 (12)
Mo3—O7—Mo4	163.12 (12)	O10—Mo4—O7	77.14 (10)
Mo2^i^—O7—Mo2	104.06 (10)	O6^i^—Mo4—O7	73.14 (10)
Mo3—O7—Mo2	97.67 (9)	O11—Mo4—O13	164.42 (12)
Mo4—O7—Mo2	97.46 (9)	O12—Mo4—O13	89.13 (12)
Mo2^i^—O7—Mo1	163.93 (13)	O10—Mo4—O13	81.58 (11)
Mo3—O7—Mo1	86.24 (8)	O6^i^—Mo4—O13	71.62 (10)
Mo4—O7—Mo1	85.80 (8)	O7—Mo4—O13	71.48 (9)
Mo2—O7—Mo1	91.98 (9)	O11—Mo4—Mo2^i^	86.16 (10)
Mo2—O9—Mo1	117.87 (14)	O12—Mo4—Mo2^i^	135.73 (11)
Mo4—O10—Mo1	116.36 (13)	O10—Mo4—Mo2^i^	118.71 (8)
Mo2—O13—Mo3^i^	108.94 (12)	O6^i^—Mo4—Mo2^i^	34.97 (7)
Mo2—O13—Mo4	110.49 (11)	O7—Mo4—Mo2^i^	41.57 (6)
Mo3^i^—O13—Mo4	103.28 (11)	O13—Mo4—Mo2^i^	78.96 (6)
O1—Mo1—O2	104.71 (15)	C3—N1—C1	109.3 (4)
O1—Mo1—O5	99.04 (13)	C3—N1—C5	125.2 (4)
O2—Mo1—O5	103.61 (14)	C1—N1—C5	125.4 (4)
O1—Mo1—O10	98.14 (13)	C3—N2—C2	109.9 (4)
O2—Mo1—O10	101.72 (14)	C3—N2—H2N	126 (3)
O5—Mo1—O10	144.48 (11)	C2—N2—H2N	125 (3)
O1—Mo1—O9	163.59 (12)	C11—N3—C9	108.5 (4)
O2—Mo1—O9	91.61 (13)	C11—N3—C8	125.1 (4)
O5—Mo1—O9	78.38 (11)	C9—N3—C8	126.1 (4)
O10—Mo1—O9	76.43 (11)	C11—N4—C10	109.8 (4)
O1—Mo1—O7	93.85 (12)	C11—N4—H4N	125 (4)
O2—Mo1—O7	161.41 (13)	C10—N4—H4N	125 (4)
			
N1—C1—C2—N2	−0.6 (6)	Mo1—O5—Mo3—O6	93.10 (15)
N1—C5—C6—C7	−179.3 (4)	Mo1—O5—Mo3—Mo2^i^	19.56 (18)
C5—C6—C7—C8	163.4 (4)	Mo2^i^—O7—Mo3—O3	−77.41 (13)
C6—C7—C8—N3	176.4 (4)	Mo4—O7—Mo3—O3	24.5 (5)
N3—C9—C10—N4	0.5 (5)	Mo2—O7—Mo3—O3	178.06 (13)
Mo3—O5—Mo1—O1	71.31 (18)	Mo1—O7—Mo3—O3	86.56 (12)
Mo3—O5—Mo1—O2	178.95 (16)	Mo2^i^—O7—Mo3—O4	92.4 (3)
Mo3—O5—Mo1—O10	−46.7 (3)	Mo4—O7—Mo3—O4	−165.6 (4)
Mo3—O5—Mo1—O9	−92.21 (16)	Mo2—O7—Mo3—O4	−12.1 (4)
Mo3—O5—Mo1—O7	−20.10 (14)	Mo1—O7—Mo3—O4	−103.6 (3)
Mo4—O10—Mo1—O1	−69.16 (17)	Mo2^i^—O7—Mo3—O5	−178.32 (12)
Mo4—O10—Mo1—O2	−176.11 (16)	Mo4—O7—Mo3—O5	−76.4 (4)
Mo4—O10—Mo1—O5	49.1 (3)	Mo2—O7—Mo3—O5	77.15 (11)
Mo4—O10—Mo1—O9	95.07 (15)	Mo1—O7—Mo3—O5	−14.35 (10)
Mo4—O10—Mo1—O7	22.57 (13)	Mo2^i^—O7—Mo3—O13^i^	18.11 (10)
Mo2—O9—Mo1—O1	−5.8 (5)	Mo4—O7—Mo3—O13^i^	120.1 (4)
Mo2—O9—Mo1—O2	−179.75 (17)	Mo2—O7—Mo3—O13^i^	−86.41 (10)
Mo2—O9—Mo1—O5	76.68 (16)	Mo1—O7—Mo3—O13^i^	−177.91 (10)
Mo2—O9—Mo1—O10	−78.08 (16)	Mo2^i^—O7—Mo3—O6	93.89 (10)
Mo2—O9—Mo1—O7	−0.15 (13)	Mo4—O7—Mo3—O6	−164.2 (5)
Mo2^i^—O7—Mo1—O1	1.4 (5)	Mo2—O7—Mo3—O6	−10.64 (8)
Mo3—O7—Mo1—O1	−83.95 (12)	Mo1—O7—Mo3—O6	−102.14 (10)
Mo4—O7—Mo1—O1	81.15 (12)	Mo4—O7—Mo3—Mo2^i^	102.0 (5)
Mo2—O7—Mo1—O1	178.50 (11)	Mo2—O7—Mo3—Mo2^i^	−104.53 (11)
Mo2^i^—O7—Mo1—O2	−175.8 (4)	Mo1—O7—Mo3—Mo2^i^	163.97 (13)
Mo3—O7—Mo1—O2	98.9 (4)	Mo2—O6—Mo3—O3	49.4 (5)
Mo4—O7—Mo1—O2	−96.0 (4)	Mo4^i^—O6—Mo3—O3	−68.0 (5)
Mo2—O7—Mo1—O2	1.3 (4)	Mo2—O6—Mo3—O4	−166.62 (16)
Mo2^i^—O7—Mo1—O5	99.7 (5)	Mo4^i^—O6—Mo3—O4	75.99 (14)
Mo3—O7—Mo1—O5	14.36 (10)	Mo2—O6—Mo3—O5	−64.60 (14)
Mo4—O7—Mo1—O5	179.46 (11)	Mo4^i^—O6—Mo3—O5	178.01 (13)
Mo2—O7—Mo1—O5	−83.20 (11)	Mo2—O6—Mo3—O13^i^	91.79 (13)
Mo2^i^—O7—Mo1—O10	−96.0 (5)	Mo4^i^—O6—Mo3—O13^i^	−25.60 (11)
Mo3—O7—Mo1—O10	178.68 (11)	Mo2—O6—Mo3—O7	13.91 (11)
Mo4—O7—Mo1—O10	−16.22 (10)	Mo4^i^—O6—Mo3—O7	−103.48 (12)
Mo2—O7—Mo1—O10	81.12 (11)	Mo2—O6—Mo3—Mo2^i^	56.15 (10)
Mo2^i^—O7—Mo1—O9	−177.0 (5)	Mo4^i^—O6—Mo3—Mo2^i^	−61.24 (9)
Mo3—O7—Mo1—O9	97.65 (10)	Mo1—O10—Mo4—O11	68.55 (18)
Mo4—O7—Mo1—O9	−97.25 (10)	Mo1—O10—Mo4—O12	176.55 (15)
Mo2—O7—Mo1—O9	0.09 (8)	Mo1—O10—Mo4—O6^i^	−55.9 (3)
Mo1—O9—Mo2—O8	−179.70 (14)	Mo1—O10—Mo4—O7	−23.21 (14)
Mo1—O9—Mo2—O6	−75.94 (15)	Mo1—O10—Mo4—O13	−96.01 (15)
Mo1—O9—Mo2—O13	77.08 (15)	Mo1—O10—Mo4—Mo2^i^	−23.58 (18)
Mo1—O9—Mo2—O7^i^	2.4 (4)	Mo2^i^—O7—Mo4—O11	79.27 (13)
Mo1—O9—Mo2—O7	0.14 (12)	Mo3—O7—Mo4—O11	−22.9 (4)
Mo1—O9—Mo2—Mo4^i^	−75.60 (17)	Mo2—O7—Mo4—O11	−176.38 (13)
Mo1—O9—Mo2—Mo3^i^	76.39 (16)	Mo1—O7—Mo4—O11	−84.92 (12)
Mo4^i^—O6—Mo2—O8	−74.26 (16)	Mo2^i^—O7—Mo4—O12	−92.7 (4)
Mo3—O6—Mo2—O8	171.55 (14)	Mo3—O7—Mo4—O12	165.2 (4)
Mo4^i^—O6—Mo2—O9	179.57 (13)	Mo2—O7—Mo4—O12	11.7 (4)
Mo3—O6—Mo2—O9	65.39 (14)	Mo1—O7—Mo4—O12	103.1 (4)
Mo4^i^—O6—Mo2—O13	65.6 (3)	Mo2^i^—O7—Mo4—O10	−179.50 (12)
Mo3—O6—Mo2—O13	−48.6 (3)	Mo3—O7—Mo4—O10	78.4 (4)
Mo4^i^—O6—Mo2—O7^i^	23.24 (12)	Mo2—O7—Mo4—O10	−75.15 (11)
Mo3—O6—Mo2—O7^i^	−90.94 (12)	Mo1—O7—Mo4—O10	16.30 (10)
Mo4^i^—O6—Mo2—O7	101.06 (13)	Mo2^i^—O7—Mo4—O6^i^	−18.44 (10)
Mo3—O6—Mo2—O7	−13.13 (10)	Mo3—O7—Mo4—O6^i^	−120.6 (4)
Mo3—O6—Mo2—Mo4^i^	−114.19 (18)	Mo2—O7—Mo4—O6^i^	85.91 (11)
Mo4^i^—O6—Mo2—Mo3^i^	24.83 (16)	Mo1—O7—Mo4—O6^i^	177.37 (11)
Mo3—O6—Mo2—Mo3^i^	−89.36 (11)	Mo2^i^—O7—Mo4—O13	−94.20 (10)
Mo3^i^—O13—Mo2—O8	75.02 (15)	Mo3—O7—Mo4—O13	163.7 (5)
Mo4—O13—Mo2—O8	−172.19 (13)	Mo2—O7—Mo4—O13	10.15 (8)
Mo3^i^—O13—Mo2—O9	−179.12 (13)	Mo1—O7—Mo4—O13	101.60 (9)
Mo4—O13—Mo2—O9	−66.32 (14)	Mo3—O7—Mo4—Mo2^i^	−102.1 (5)
Mo3^i^—O13—Mo2—O6	−64.9 (3)	Mo2—O7—Mo4—Mo2^i^	104.35 (11)
Mo4—O13—Mo2—O6	47.9 (3)	Mo1—O7—Mo4—Mo2^i^	−164.19 (12)
Mo3^i^—O13—Mo2—O7^i^	−22.44 (12)	Mo2—O13—Mo4—O11	−38.2 (5)
Mo4—O13—Mo2—O7^i^	90.36 (12)	Mo3^i^—O13—Mo4—O11	78.2 (5)
Mo3^i^—O13—Mo2—O7	−100.24 (12)	Mo2—O13—Mo4—O12	167.31 (15)
Mo4—O13—Mo2—O7	12.55 (10)	Mo3^i^—O13—Mo4—O12	−76.32 (14)
Mo3^i^—O13—Mo2—Mo4^i^	−23.85 (15)	Mo2—O13—Mo4—O10	65.99 (13)
Mo4—O13—Mo2—Mo4^i^	88.95 (11)	Mo3^i^—O13—Mo4—O10	−177.64 (12)
Mo4—O13—Mo2—Mo3^i^	112.80 (16)	Mo2—O13—Mo4—O6^i^	−91.00 (13)
Mo2^i^—O7—Mo2—O9	179.05 (13)	Mo3^i^—O13—Mo4—O6^i^	25.37 (11)
Mo3—O7—Mo2—O9	−86.59 (12)	Mo2—O13—Mo4—O7	−13.19 (11)
Mo4—O7—Mo2—O9	85.91 (12)	Mo3^i^—O13—Mo4—O7	103.18 (11)
Mo1—O7—Mo2—O9	−0.12 (10)	Mo2—O13—Mo4—Mo2^i^	−55.58 (10)
Mo2^i^—O7—Mo2—O6	−81.79 (12)	Mo3^i^—O13—Mo4—Mo2^i^	60.79 (8)
Mo3—O7—Mo2—O6	12.57 (10)	N2—C3—N1—C1	0.0 (6)
Mo4—O7—Mo2—O6	−174.94 (12)	C4—C3—N1—C1	−178.8 (6)
Mo1—O7—Mo2—O6	99.04 (10)	N2—C3—N1—C5	−177.8 (4)
Mo2^i^—O7—Mo2—O13	81.18 (12)	C4—C3—N1—C5	3.4 (8)
Mo3—O7—Mo2—O13	175.54 (12)	C2—C1—N1—C3	0.4 (6)
Mo4—O7—Mo2—O13	−11.97 (10)	C2—C1—N1—C5	178.2 (4)
Mo1—O7—Mo2—O13	−97.99 (10)	C6—C5—N1—C3	89.7 (6)
Mo2^i^—O7—Mo2—O7^i^	0.0	C6—C5—N1—C1	−87.7 (6)
Mo3—O7—Mo2—O7^i^	94.36 (11)	N1—C3—N2—C2	−0.4 (6)
Mo4—O7—Mo2—O7^i^	−93.14 (11)	C4—C3—N2—C2	178.5 (6)
Mo1—O7—Mo2—O7^i^	−179.17 (14)	C1—C2—N2—C3	0.6 (6)
Mo2^i^—O7—Mo2—Mo4^i^	−46.75 (8)	N4—C11—N3—C9	0.1 (5)
Mo3—O7—Mo2—Mo4^i^	47.61 (7)	C12—C11—N3—C9	−179.8 (5)
Mo4—O7—Mo2—Mo4^i^	−139.89 (7)	N4—C11—N3—C8	−173.5 (4)
Mo1—O7—Mo2—Mo4^i^	134.08 (6)	C12—C11—N3—C8	6.6 (7)
Mo2^i^—O7—Mo2—Mo3^i^	45.79 (8)	C10—C9—N3—C11	−0.4 (5)
Mo3—O7—Mo2—Mo3^i^	140.15 (7)	C10—C9—N3—C8	173.1 (4)
Mo4—O7—Mo2—Mo3^i^	−47.35 (7)	C7—C8—N3—C11	−138.3 (5)
Mo1—O7—Mo2—Mo3^i^	−133.38 (6)	C7—C8—N3—C9	49.2 (6)
Mo1—O5—Mo3—O3	−72.93 (18)	N3—C11—N4—C10	0.2 (5)
Mo1—O5—Mo3—O4	179.01 (15)	C12—C11—N4—C10	−179.9 (4)
Mo1—O5—Mo3—O13^i^	50.0 (3)	C9—C10—N4—C11	−0.4 (5)
Mo1—O5—Mo3—O7	20.83 (14)		

Symmetry codes: (i) −*x*+1, −*y*+1, −*z*+1.

### Hydrogen-bond geometry (Å, °)

**Table:**

*D*—H···*A*	*D*—H	H···*A*	*D*···*A*	*D*—H···*A*
N2—H2N···O9^ii^	0.87 (3)	2.37 (4)	3.031 (5)	134 (4)
N2—H2N···O10^ii^	0.87 (3)	2.20 (2)	3.001 (5)	153 (5)
N4—H4N···O12^iii^	0.86 (3)	2.39 (4)	3.052 (5)	134 (4)
N4—H4N···O4^iv^	0.86 (3)	2.12 (3)	2.873 (4)	146 (5)

Symmetry codes: (ii) −*x*+1, −*y*+1, −*z*; (iii) *x*−1, *y*−1, *z*+1; (iv) −*x*, −*y*, −*z*+2.
